# A Cross-Lagged Panel Analysis of the Relationship Between Loneliness and Depression

**DOI:** 10.1093/geroni/igaa057.1989

**Published:** 2020-12-16

**Authors:** Jillian Minahan, Karen Siedlecki

**Affiliations:** 1 Fordham University, Bronx, New York, United States; 2 Fordham University, New York, New York, United States

## Abstract

Loneliness and depression have similar psychological features but are theoretically and statistically distinct (Hawkley et al., 2008). Most research has examined a unidirectional relationship between loneliness and depression (Cacioppo et al., 2006). Cacioppo et al. (2010), however, found that loneliness predicted subsequent depression, but depression did not predict subsequent loneliness. We extended this work by examining the reciprocal relationship between loneliness and depression in 1,560 healthy adults (ages ranging from 18-95 years at baseline) from the Virginia Cognitive Aging Project using cross-lagged panel analysis across three time points. Depression more strongly predicted subsequent loneliness (βs= 0.16 and 0.23, p < .05) than vice versa. These findings are consistent with the cognitive discrepancy theory, suggesting that affective processing, like depression, can negatively impact one’s perception of their social environment (Burholt & Scharf, 2014). Our findings highlight the need for increased longitudinal research examining the relationships among indices of well-being across adulthood.

